# Interhemispheric Acute Subdural Hematoma: A Distinct Entity of Subdural Hematoma

**DOI:** 10.14740/jocmr2427w

**Published:** 2015-12-28

**Authors:** Ozgur Sogut, Mehmet Yigit, Kenan Ahmet Turkdogan, Eda Yigit, Bedia Gulen, Ertan Sonmez, Onur Kaplan, Huseyin Toprak

**Affiliations:** a Department of Emergency Medicine, Haseki Training and Research Hospital, Istanbul, Turkey; b Department of Emergency Medicine, School of Medicine, Aydin Menderes University, Aydin, Turkey; c Department of Emergency Medicine, Sisli Etfal Training and Research Hospital, Istanbul, Turkey; d Department of Emergency Medicine, School of Medicine, Bezmialem Vakif University, Istanbul, Turkey; e Department of Radiology, School of Medicine, Bezmialem Vakif University, Istanbul, Turkey

## To the Editor

**Figure 1:**
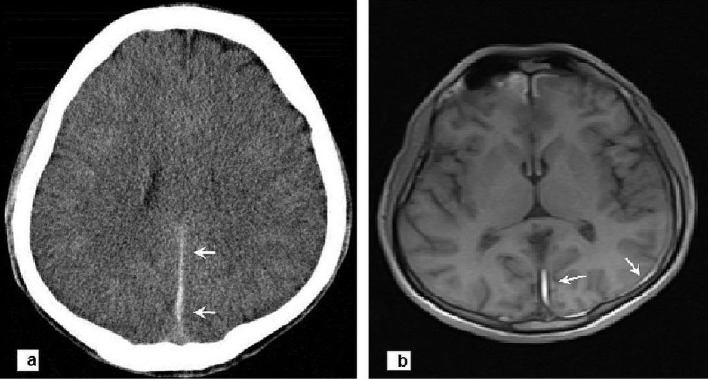
(a) Axial CT scan of brain performed 4 h after the accident revealing a small left-sided acute interhemispheric subdural hematoma, which was posteriorly located (arrows). (b) Cranial MRI showing minimal ASH in the posterior interhemispheric fissure (arrows) with subacute subdural hematoma in the left parieto-occipital cortex and occipital regions (arrows).

We wish to notify the readers on a rare case of interhemispheric acute subdural hematoma (ASH) who presented to our emergency department (ED) with a falx syndrome of contralateral hemiparesis in lower extremity and severe headache. A 25-year-old previously healthy man was involved in a traffic accident and presented to our ED with complaints of repeated vomiting and severe headache. On arrival to the ED, he had an initial Glasgow coma scale (GCS) of 14 (opening her eyes with verbal stimuli, obeys commands, orientated) and normal vital signs (blood pressure: 130/85 mm Hg; heart rate: 86 bpm). No other injuries were noted. Neurological examination revealed mild weakness of the right lower extremity (strength score: 4/5). His routine blood tests, including complete blood counts, prothrombin time, and thromboplastin time were unremarkable. The plain films of the skull were normal. Non-enhanced cranial (CT) scan performed 4 h after the accident revealed a small left-sided acute interhemispheric subdural hematoma, which was posteriorly located (Fig. 1a). Magnetic resonance imaging (MRI) of the brain during the first 24 h after admission confirmed minimal ASH in the posterior interhemispheric fissure with subacute subdural hematoma in the left parieto-occipital cortex and occipital regions (Fig. 1b). Considering the clinical condition of the patient, surgery was not planned by the neurosurgical department but the patient was kept under observation in the ED. He was discharged home 48 h after the trauma with a normal neurological examination and mild intermittent headaches.

Interhemispheric ASH is a relatively uncommon type of ASH because of their unusual location [1]. They usually occur in patients with bleeding disorders and are associated with trauma in the majority of cases [2]. Computed tomography (CT) and MRI are important neuroradiological techniques for the accurate diagnosis of interhemispheric ASH [1, 2]. Although interhemispheric ASH is a rare form of subdural hematoma, early diagnosis and treatment is of great importance because of its emergent condition [2, 3]. CT and MRI are important diagnostic tools, as it was in this case. MRI may have the advantage rather than CT due to the absence of beam-hardening and multiplanar imaging. The best management modality for interhemispheric ASH depends upon the neurologic status on admission and clinical course [2]. If the patient is neurologically stable, conservative treatment is generally preferred. Surgical treatment is reserved in patients with disturbances of consciousness and for patients with progressive neurological deterioration [3].
